# γδ T cells as sensors of ectopically expressed intracellular proteins

**DOI:** 10.1038/s41423-025-01269-8

**Published:** 2025-03-19

**Authors:** Dieter Kabelitz, Jaydeep Bhat

**Affiliations:** 1 Institute of Immunology, University of Kiel and University Hospital Schleswig-Holstein Campus Kiel, Kiel, Germany; 2 Next Generation Sequencing Core Facility, German Cancer Research Centre, Heidelberg, Germany

**Keywords:** Immunology, Oncology

γδ T cells play important roles in the immunosurveillance of infection, cellular stress, and malignant transformation. In contrast to conventional αβ T cells, the T-cell receptor (TCR) of γδ T cells does not recognize antigen-derived peptides in the context of MHC class I or class II molecules but rather senses stress-inducible surface molecules and microbe-derived or endogenous metabolites. You et al. discovered that certain nuclear proteins, such as nucleolin, can be ectopically expressed on the surface of tumor cells and then serve as ligands for human γδ T cells [1]. The demonstration that γδ T cells recognize intracellular proteins that are ectopically expressed on the surface of somatic cells represents a new paradigm for the immunosurveillance function of γδ T cells.

**Fig. 1:**
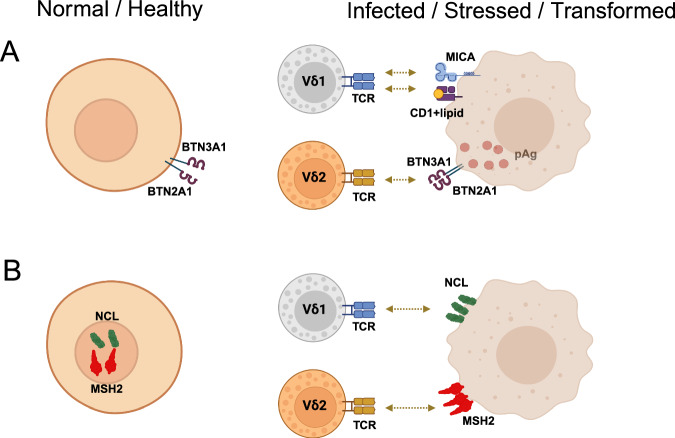
γδ T cells differentiate between healthy and infected/stressed/transformed cells. **A** *Left*: Healthy cells express BTN2A1 and BTN3A1 but do not activate Vδ2 T cells because they do not produce phosphoantigens (pAgs). Similarly, healthy cells usually do not express MICA. *Right*: Upon infection or stress, cells upregulate MICA and CD1 isoforms, which can bind lipids, both of which are direct ligands for the Vδ1 TCR. Stressed/infected/transformed cells also produce pAg, which activates Vδ2 T cells in a BTN2A1/3A1-dependent manner. **B** *Left*: Nucleolin (NCL) and the DNA mismatch repair protein mutS homolog 2 (MSH2) are expressed in the nucleus and do not activate γδ T cells. *Right:* Under stress, NCL and MSH2 can be ectopically expressed on the cell surface and then activate Vδ1 (NCL) and Vδ2 (MSH2) γδ T cells. Figure created in https://BioRender.com

In addition to αβ T cells and B cells, γδ T cells constitute a third lymphoid lineage that expresses clonally rearranged antigen receptor genes. While the number of available genes coding for variable parts of the TCR γ and δ chains is small compared to the αβ TCR, the potential γδ TCR repertoire is large. This notwithstanding, there is a striking skewing of the γδ T-cell subset distribution in different locations. In human peripheral blood, γδ T cells expressing Vγ9 paired with Vδ2 (termed Vδ2 hereafter) are predominant, whereas Vδ1 T cells expressing various Vγ chains (termed Vδ1) are more abundant in epithelial tissues [2]. A major difference between conventional αβ T cells and γδ T cells is related to the ligands recognized by the respective TCR. In contrast to αβ T cells, the vast majority of γδ T cells do not recognize peptides presented by MHC class or class II molecules, which is in line with their MHC-independent mode of activation [2]. Ligands that have been verified to directly bind to the human γδ TCR include endogenous and microbial lipids bound to CD1 isoforms and stress-inducible molecules such as the endothelial protein C receptor (EPCR) and MHC class I related chain A (MICA) [2–6]. Moreover, a unique and selective pattern of activation of human Vδ2 T cells by microbial or endogenous pyrophosphates has been described in recent years. The activation of Vδ2 T cells by such “phosphoantigens” (pAgs) completely depends on the presence of transmembrane molecules of the butyrophilin family, specifically BTN2A1 and BTN3A1. pAg produced by bacteria (e.g., *M. tuberculosis*) or endogenously overproduced in tumor cells bind to the intracellular domain of BTN3A1 and induce a conformational change in the extracellular BTN3A1/2A1 complex, which then directly activates the γδ TCR [7]. In all instances, γδ T cells are not activated by healthy/normal cells (even though they express BTN2A1 and BTN3A1) but respond to infection and stress- or transformation-induced cellular alterations (Fig. 1A).

In search of protein antigens that are possibly recognized by the human γδ TCR, You et al. synthesized peptides derived from the conserved regions in the TCR CDR3δ sequences of two tumor-infiltrating γδ TCRs, GTM (Vδ1) and OT3 (Vδ2). These peptides were shown to bind to various tumor cells via immunohistochemistry and flow cytometry. To support the notion that the CDR3δ regions of the two specific TCRs derived from tumor-infiltrating γδ T cells can recognize tumor cells, chimeric antigen receptor (CAR) constructs with single chain Vγ9-Vδ2 (OT3) and Vγ4-Vδ1 (GTM) as their extracellular domains were generated. CAR-T cells expressing these constructs recognized and killed various tumor cell lines. Next, they generated soluble molecules that could be used for screening for binding to proteins via human proteome arrays. To this end, they generated a GTM-grafted Vγ4 Vδ1-Fc fusion protein and a GTM-grafted soluble γδ TCR as probes for the Vδ1 TCR and a biotinylated OT3 peptide as a probe for the Vδ2 TCR. Among the 21,000 proteins in the proteome microarray, only 13 bound specifically to the GTM-Vδ1 probes, and only three bound to the OT3-Vδ2 peptide. Quite surprisingly, all identified potential TCRγδ ligands were of intracellular origin.

In the next part of their study, the authors went on to verify the functional significance of some of the identified proteins as potential ligands for the human γδ TCR. They focused on nucleolin (NCL), a conserved RNA-binding protein involved in multiple cellular processes, including chromatin remodeling and nucleocytoplasmic transport. NCL is localized in the nucleus but can be translocated to the cell membrane in tumor cells and under stress conditions [8]. In their study, You et al. reported that NCL is upregulated in a variety of tumors and can also be expressed on the surface of different tumor cell lines. Furthermore, they also reported the upregulation of NCL in an in vitro stress model in which DNA damage is induced by menadione. A critical question of course is whether the NCL protein can directly bind to the GTM-Vδ1 TCR. There are different strategies by which the direct interaction of a potential ligand with a soluble TCR can be verified, such as Biacore surface plasmon resonance and cocrystallization. The authors were unable to demonstrate direct binding via microscale thermophoresis but were able to show the interaction of the NCL-Fc fusion protein and GTM via coimmunoprecipitation. Furthermore, the recombinant NCL protein activated Vδ1 T cells in vitro, as demonstrated by the upregulation of the activation markers CD69 and CD25 and their proliferative activity. To validate the potential role of ectopically expressed NCL protein as an antigen for tumor-reactive γδ T cells, the authors generated CAR constructs on the basis of specific anti-NCL antibodies, where Vγ and Vδ fragments were replaced by VL and VH fragments of the anti-NCL antibody. These NCL-γδ TCR CAR-T cells exhibited potent cytotoxicity in vitro against several NCL-expressing tumor cells. More importantly, they also demonstrated antitumor efficacy in vivo upon transfer into immunodeficient tumor-bearing mice.

In previous studies, another nuclear protein, the DNA mismatch repair protein mutS homolog 2 (MSH2), was identified as a ligand for Vδ2 γδ T cells, which triggers cytotoxic responses when it is ectopically expressed on the surface of tumor cells [9]. In the present study, they also identified the cytoplasmic enzyme transglutaminase 1 (TGM1) as a ligand for Vδ2, which can again be ectopically expressed on tumor cells [1]. Taken together, the findings of the study by You et al. [1] support the new paradigm that γδ T cells can sense nuclear (and cytoplasmic) proteins when these proteins are ectopically expressed upon cellular stress or malignant transformation (Fig. 1B). From a broader perspective, it thus appears that γδ T cells can differentiate between normal/healthy and damaged cells by recognizing proteins that are hidden within healthy cells and are ectopically expressed on the cell surface upon cellular stress. These new findings broaden our understanding of the intricate immunosurveillance function of γδ T cells. In essence, γδ T cells act whenever they sense abnormalities in their cellular neighborhood. This may be due to the upregulation of stress-induced expression of cell surface molecules such as MICA, increased pAg production, or ectopic surface expression of intracellular proteins (Fig. 1). Furthermore, γδ T cells also express various activating and inhibitory NK receptors, which additionally tune the activation and effector function of γδ T cells. Owing to their HLA independence, these features make γδ T cells highly attractive as effector cells for cellular immunotherapy, especially in an allogeneic setting [6, 9–11].
